# Call-Fleming Syndrome (Reversible Cerebral Artery Vasoconstriction) and Aneurysm Associated with Multiple Recreational Drug Use

**DOI:** 10.1155/2013/729162

**Published:** 2013-02-10

**Authors:** Doniel Drazin, Michael J. Alexander

**Affiliations:** Department of Neurosurgery, Cedars-Sinai Medical Center, 8631 West Third Street, Los Angeles, CA 90048, USA

## Abstract

Drug abuse represents a significant health issue. Evidence suggests that recreational drug use has a direct effect on the cerebral vasculature and is of greater concern in those with undiagnosed aneurysms or vascular malformations. The authors report a case of thunderclap headache with a negative head CT and equivocal lumbar puncture after a drug-fueled weekend. The patient underwent diagnostic cerebral angiogram which demonstrated multisegmental, distal areas of focal narrowing of the middle, anterior, posterior, and posterior inferior cerebral artery and an incidental aneurysm. It is often difficult to determine the exact origin of symptoms; thus we were left with a bit of a chicken or the egg debate, trying to decipher which part came first. Either the aneurysm ruptured with associated concomitant vasospasm or it is a case of Call-Fleming syndrome (reversible cerebral artery vasoconstriction) with an incidental aneurysm. The authors proposed their management and rationale of this complex case.

## 1. Case Description 

**Figure 1:**
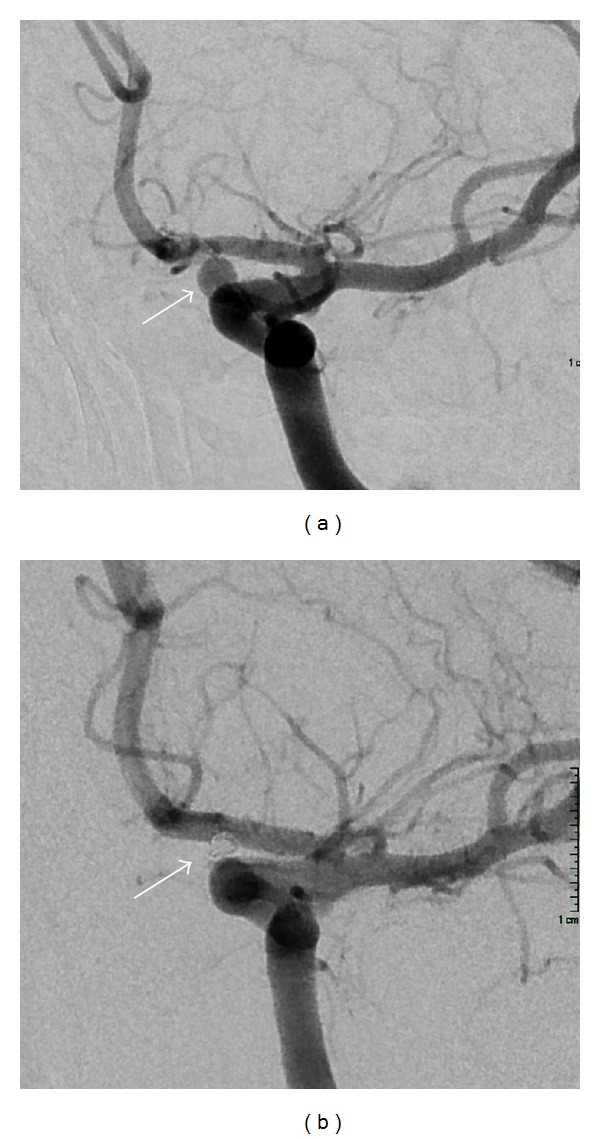
(a) Preoperative and (b) postoperative embolization images of the carotid ophthalmic aneurysm.

**Figure 2:**
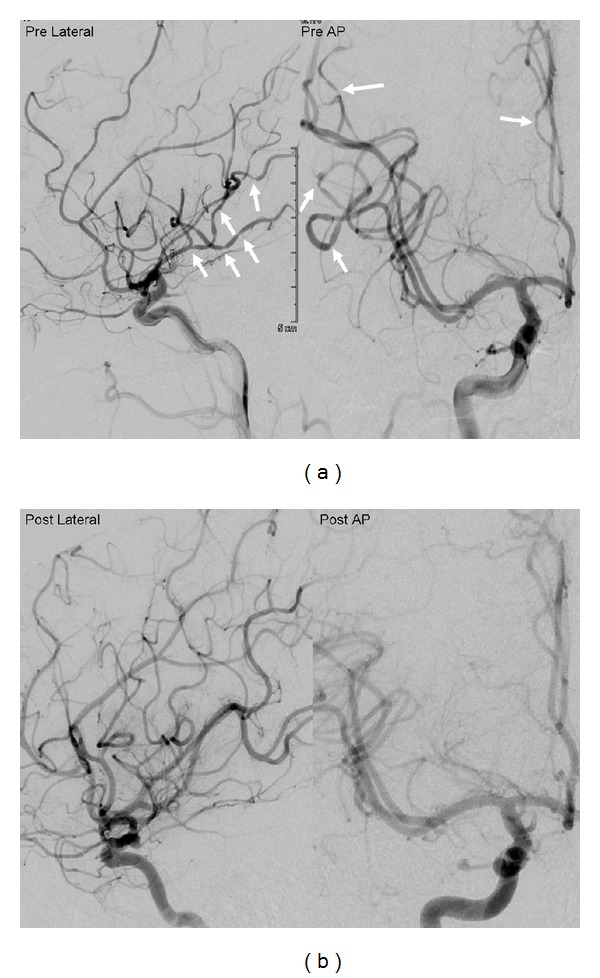
(a) Admission DSA of the right ICA shows multivessel vasoconstrictions. (b) Three-month follow-up DSA of the right ICA shows reversal of the diffuse vasoconstrictions. DSA: digital subtraction angiography. ICA: Internal carotid artery.

The authors report a reversible case of stimulant-related diffuse cerebral artery vasoconstriction, thunderclap headache, and aneurysm. A 23-year-old woman presented to the emergency department three days after recreational drug use (toxicology positive for methamphetamines, ecstasy, and marijuana) with thunderclap headache, vomiting, and seizure. On examination, she complained of nausea, blurry vision, photophobia, and severe headache unrelieved with medication. A subsequent head CT demonstrated no clear evidence of subarachnoid hemorrhage (SAH). Her lumbar puncture (LP), however, demonstrated over 200 red blood cells which remained persistent on the final tube, with equivocal xanthochromia. Her CT angiogram revealed evidence of a 2.7 mm left carotid ophthalmic aneurysm (Figure 1). The patient underwent a diagnostic cerebral angiogram which showed diffuse narrowing of the middle, anterior, posterior, and posterior inferior cerebral artery branches consistent with drug-induced vasculopathy (Figure 2) [1, 2].

## 2. Discussion

It is often difficult to determine the exact origin of symptoms. In Call-Fleming syndrome (CFS), SAH is usually minimal and only present in up to 1 in 4 cases [3–5]. Based on the clinical presentation and LP findings, our patient could have had an SAH, and the angiographic findings could have been that of a related vasospasm. Our working diagnosis was that the patient had drug-induced CFS and that the aneurysm was most likely incidental. Since the CT was negative and the patient's spasm included multiple cerebral vessels and was more segmental and not diffuse, CFS seemed the most likely diagnosis. 

Management of a cerebral aneurysm in the setting of CFS presents complexities. Endovascular treatment through constricted segments could result in ineffective occlusion of the ruptured aneurysm and a high rate of complications. Spasm can lead to flow arrest in the vessel during microcatheter access. Treatment of spasm prior to coiling risks an aneurysm rupture during vasodilation of the artery [6].

Spasm presents a challenge for both surgical clipping and endovascular treatment. Several studies of microsurgical aneurysm clipping during vasospasm report high morbidity and poor outcomes [7–10]. Although there are limited studies on endovascular treatment of CFS, the literature documents concomitant vasospasm as safe and efficacious for endovascular treatment [11]. Simultaneous coil embolization of a ruptured aneurysm and endovascular treatment of associated vasospasm have been described with some degrees of immediate safety [11], and preliminary multicenter results have shown its efficacy with encouraging long-term angiographic and clinical outcomes [12].

SAH from the aneurysm was in the differential diagnosis (since symptoms began a few days earlier). Since ruptured aneurysms have such a high rate of early rebleed, we opted to treat the aneurysm acutely, and our patient underwent successful coil embolization of the aneurysm with no complications. Her follow-up angiogram at three months showed reversal of the arterial vasoconstrictions (Figure 2). Some CFS patients report headache relief from calcium channel blockers, but our patient's headaches were relieved with simple analgesic [13].

Stimulants, such as cocaine, methamphetamines, and ecstasy, have been documented to cause intracerebral, intraventricular, or subarachnoid hemorrhage [1]. The hypothesis is that stimulant use causes sympathetic hyperstimulation which results in significant hypertension and, in turn, leads to cerebral vasoconstriction [1]. Reversible cerebral vasoconstriction syndrome, also known as Call-Fleming syndrome, has been reported to be associated with sympathomimetics and drugs that modulate the dopamine and serotonin systems [2].

Stimulant drug use can result in reversible cerebral artery vasoconstriction. However, in the setting of a concomitant cerebral aneurysm, management may be more complex.
